# Localization of mandibular foramen – a comparison between dry bones and orthopantomogram

**DOI:** 10.25122/jml-2022-0007

**Published:** 2022-05

**Authors:** Ravdeep Kaur, Rajan Kumar Singla, Ravikant Sharma, Sanju Singla

**Affiliations:** 1. Department of Anatomy, Government Medical College, Amritsar, Punjab, India; 2. Department of Anatomy, Government Medical College, Patiala, Punjab, India; 3. Department of Oral Maxillofacial Surgery, Luxmi Bai Institute of Dental Sciences, Patiala, Punjab, India

**Keywords:** panoramic radiograph, mandibular foramen, inferior alveolar nerve block, magnification, dry mandible

## Abstract

An inferior alveolar nerve block is a usual practice by a dental practitioner. Panoramic radiography is a widely used technique in dentistry to get a clear and comprehensive view before planning any treatment. The study aimed to compare the morphometric localization of mandibular foramen (MF) on dry bones and orthopantomogram. The study was designed in two phases: a morphometric study on dry human mandibles (phase I) and orthopantomograms of the same dry human mandibles (phase II). The study materials were 200 dry north Indian human mandibles belonging to unknown sex obtained from the Department of Anatomy. Descriptive statistics, including range, mean±standard deviation, paired t-test to compare dry bones and orthopantomogram, Pearson's correlation coefficient, and measurement error, were used. T-test was applied separately to compare the right and left sides of dry bones. The distance of mandibular foramen from the posterior border and lower border is shorter on the right side than on the left. Its distance from the anterior border and the mandibular notch was greater on the right side. On panoramic radiographs, the distance of MF from nearby anatomical landmarks on the mandible was highly unreliable except for the mandibular notch. Our findings demonstrate a statistically significant difference between distances on dry bone and OPG but no statistically significant difference between MF-notch on both sides and MF-AB on the right side. As a result, a surgeon can rely upon a mandibular notch to locate mandibular foramen during clinical procedures. Magnification is an inbuilt property of OPG; for precise localization of MF, it is advisable to proceed with advanced three-dimensional techniques to protect viable anatomical structures.

## INTRODUCTION

The mandibular foramen is present on the medial aspect of the ramus, and it transmits inferior alveolar nerves and vessels. During an inferior alveolar nerve block, it is important to administer an anaesthetic agent in the inferior alveolar nerve without violating inferior alveolar vessels [1]. In a procedure like an osteotomy, where the ramus is assessed and cut without visualization of the medial surface, this neurovascular bundle is vulnerable [2].

Orthopantomogram (OPG) is an extra-oral imaging technique. It is useful to evaluate diagnostic problems which require broad coverage [3]. Therefore, this study was conducted to compare the differences between dry bones with their OPG.

## MATERIAL AND METHODS

The materials for the current study comprised of 200 dry north Indian human mandibles belonging to unknown sex, obtained from the Anatomy Department of Government Medical College, Amritsar. Orthopantomograms of these dry mandibles were taken. Anatomical landmarks were used to measure various distances and parameters on dry bone as well as on orthopantomograms.

### Inclusion criteria

Intact and well-formed mandibles varying from dentulous to partially dentulous mandibles.

### Exclusion criteria

Fractured, damaged, and mutilated mandibles were excluded from the study. The present study was designed in two phases:

Phase I (dry bone phase) consisted of a morphometric study of dry human mandibles. Phase II (radiographic phase) consisted of orthopantomograms of the same dry human mandibles. Mandibles with mandibular foramen not visible on OPG were dropped out from comparison.

### Methodology in phase l

All the mandibles were serialised from numbers 1 to 200. A vernier caliper with a least count of 0.01 mm was used to measure the distances on dry bones.

### Methodology in phase Il

Digital panoramic radiographs of the same dry mandibles were taken. Each dry mandible was centered in the focal trough of the digital panoramic machine by a reference line parallel to the symphysis menti to keep the method of study consistently standardized. OPG were taken by ADVAPEX- machine. Exposure parameters were: anode voltage: 65 kvp, tube current: 10 mA, exposure time: 14 seconds, magnification index given by manufacturer: 1.2. After exposure parameters, radiographs were stored on a computer. Later on, all morphometric measurements were taken with the software ImageWorks-DICOM CD Viewer and recorded on the pre-designed proforma. To assure an accurate outline of key identifiable anatomical structures in an OPG, guidance of subject experts was taken.

### Parameters on dry bones and OPG

The reference point (F) for mandibular foramen (MF) was taken as the lowest point of the lower border of the mandibular foramen, where it merges with the lingula [4]. The following parameters were measured on dry bones as well as on OPG.

**Figure 1:**
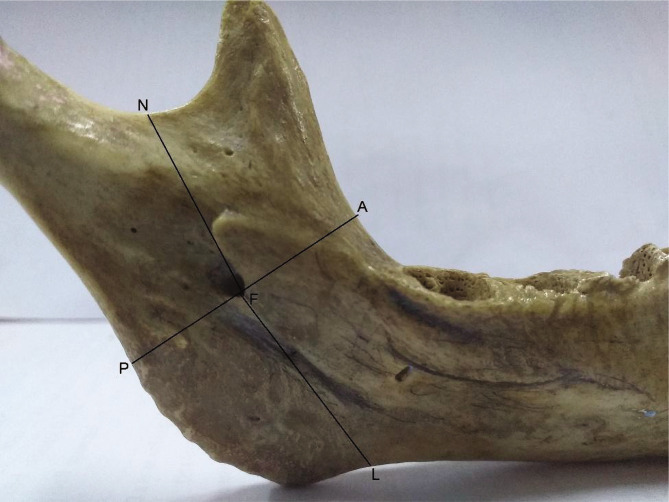
Metric measurements of MF from anatomical landmarks on dry bones. Point F – The reference point of the mandibular foramen; Line AF – Distance from the reference point of MF to the anterior border of the ramus; PF – Distance between the lower border of the mandibular foramen (reference point) to the nearest point on the posterior border of the ramus; NF – Distance between the lower border of the mandibular foramen and mandibular notch; LF – Distance from the lower border of the mandibular foramen (reference point) to the lower border of the mandible.

**Figure 2:**
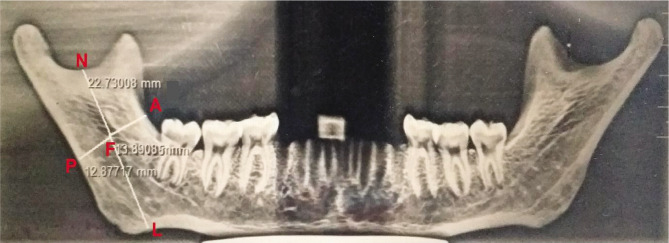
OPG showing metric measurements of MF from landmarks.Point F: The reference point of the mandibular foramen. Line AF – distance from the reference point of MF to the anterior border of the ramus. PF – Distance between the reference point of MF to the posterior border of the ramus. NF – Distance reference point of MF to mandibular notch. LF – Distance from the reference point of MF to the lower border of the mandible.

1. MF – posterior border of the ramus (PB): distance between the lower border of mandibular foramen (reference point) and the nearest point on the posterior border of the ramus [4–7] (Line FP in Figures 1 and 2).
2. MF – anterior border of the ramus (AB): distance between the lower border of the mandibular foramen and the anterior border of the ramus [5–7] (Line FA in Figures 1 and 2).
3. MF – mandibular notch (MN): distance between the lower border of the mandibular foramen and mandibular notch [7–9] (Line FN in Figures 1 and 2).
4. MF – lower border of the mandible (LB): Perpendicular distance between the lower border of mandibular foramen (reference point) and lower border of the mandible [7, 8] (Line FL in Figures 1 and 2).

The perpendicular distance was taken perpendicular to the standard basal plane. A standard basal plane is a horizontal plane with which the lower border of the mandible makes maximum contact when vertical pressure is applied to the region of the second molar tooth [4].

### Statistical analysis

Mean and standard deviation was calculated. A student t-test was applied to compare data obtained from dry bones and OPG. The P-value of comparison between dry bone and OPG was recorded, and if it was less than 0.05, it was considered statistically significant. The range was recorded to know the minimum and maximum distance of MF from nearby anatomical landmarks. Pearson's correlation coefficient was applied to analyze the strength of association between both techniques. SPSS 18 was used for statistical analysis. Since distortion and magnification are inbuilt properties of every panoramic machine, the following formula was used to evaluate magnification [10]:


$$\text{Measurement Error (\%s) =}\frac{MBS\text{ on OPG - MBS on dry bone}}{\text{MBS on dry bone}}\times 100$$

MBS – mean of bone size.

## RESULTS

When MF boundaries were ill-defined, it was considered invisible on OPG. In the present study, out of 200 OPG, bilateral MF was invisible on 8 radiographs, unilateral on the right side in 4 OPG, and on the left side in 5 OPG. Pearson's correlation coefficient was calculated, and values showed a positive correlation between dry bones and OPG. The highest correlation was found in MF to notch on the left side, followed by MF to the lower border of the mandible. The lowest correlation was found in MF to the anterior border of the ramus of the mandible on the left side. All the values obtained on dry bone and OPG were tabulated and subjected to statistical analysis. The distance of MF from the lower border of the mandible and the posterior border of the ramus showed a highly statistically significant difference (p≤0.0001*, 0.0001*, respectively) in comparing dry bones and OPG.The t-test showed no statistically significant difference in MF-MN distance between dry bones and OPG on both sides (RT, p=0.62, LT, p=0.07). Results of statistical analysis are compiled in Tables 1 and 2. The comparison of results from this study with accessible literature is compiled in Tables 3 and 4.

**Table 1:** Statistical results of dry bones and OPG.

Parameters		Mean±SD (mm)		Range (mm)		P-value (comparison of dry and OPG)		Correlation coefficient (r)	
		RT	LT	RT	LT	RT	LT	RT	LT
**MF-LB**	Dry	23.85±4.21	24.81±4.77	13.34–36.81	14.20–41.53	<0.0001*	<0.0001*	.858	.872
	OPG	27.26±4.37	27.99±4.93	16.32–38.90	15.78–43.25				
**MF-PB**	Dry	13.51±2.10	14.16±2.27	9.12–21.26	8.87–19.83	0.0001*	0.0002*	.692	.770
	OPG	14.46±2.46	15.11±2.58	8.34–20.78	6.99–25.39				
**MF-Notch**	Dry	23.44±3.86	23.05±3.99	14.10–37.76	13.36–38.29	0.62	0.07	.832	.882
	OPG	23.65±4.50	23.83±4.51	14.11–40.94	12.28–38.41				
**MF-AB**	Dry	16.41±2.42	16.18±2.47	11.09–24.84	9.81–26.90	0.35	0.03*	.790	.103
	OPG	16.51±2.47	15.95±2.59	8.90–24.51	9.53–26.01				

MF-LB – Mandibular foramen to lower border of mandible; MF-PB – Mandibular foramen to posterior border of ramus; MF-Notch – Mandibular foramen to mandibular notch; MF-AB – Mandibular foramen to anterior border of ramus of mandible. P value – * is significant.

**Table 2:** Error of magnification.

Parameters	Error of magnification %	
	RT	LT
**MF-LB**	14.30	12.82
**MF-PB**	7.03	6.71
**MF-Notch**	0.86	3.38
**MF-AB**	0.60	-1.42

MF-LB – Mandibular foramen to lower border of mandible; MF-Notch – Mandibular foramen to posterior border of ramus; MF-PB – Mandibular foramen to mandibular notch; MF-AB – Mandibular foramen to anterior border of ramus of mandible.

**Table 3:** Data on dry bones available in accessible literature regarding measured parameters.

Author		MF-LB		MF-PB		MF-Notch		MF-AB	
		RT	LT	RT	LT	RT	LT	RT	LT
**Gop *al*krishna et *al***. [5]	Dry	-	-	12.34±3.10	13.51±3.92	21.23±4.56	21.16±3.12	14.63±3.16	15.31±3.11
**Gupta et *al***. [6]	Dry	-	-	14.31±1.82	14.39±1.79	-	-	18.9±2.14	18.88±2.34
**L *al*itha et *al***. [7]	Dry	27.41±4.16	26.76±4.14	14.05±2.19	13.90±2.35	20.14±2.5	19.85±3.15	16.52±2.25	17.77±2.51
**Padmavathi et *al***. [8]	Dry	25.0±3.2	24.8±3.3	-	-	-	22.3±3.4	-	-
**Nivedha et *al***. [9]	Dry	21.06±5.23	20.73±5.23	11.42±2.02	12.94±5.64	15.54±2.70	15.39±2.89	16.07±2.80	16.31±3.24
**Oguz and Bozkir** [13]	Dry	-	-	14.09	14.37	22.37	22.17	16.90	16.78
**Thangavelu et *al***. [14]	Dry	27.62±4.20	27.30±4.19	14.31±1.82	14.39±1.79	-	-	18.9±9.14	18.88±2.34
**Kumari S et *al***. [15]	Dry	-	-	10.21	10.28	20.48	20.15	16.00	16.27
**Prado et *al***. [16]	Dry	-	-	14.20	13.00	23.60	23.10	19.20	18.80
**Hoque et *al***. [17]	Dry	-	-	14.14	14.04	22.29	22.18	16.34	16.27
**Jain et *al***. [18]	Dry	-	-	12.31±2.49	11.75±2.47	17.41±3.22	18.01±3.44	16.88±2.43	17.33±2.24
**Reddy et *al***. [19]	Dry	25.56±4.33	25.31±4.21	-	-	24.03±6.83	23.96±6.62	-	-
**Patil et *al***. [20]	Dry	-	-	13.33±1.57		23.67±3.45		24.36±2.31	
**Nagraj et *al***. [23]	Dry	-	-	-	-	44.82±4.01	44.12±4.15	-	-
**Present study**	Dry	23.85±4.21	24.81±4.77	13.51±2.10	14.16±2.27	23.44±3.86	23.05±3.99	16.41±2.42	16.18±2.47
	OPG	27.26±4.37	27.19±4.93	14.46±2.46	15.11±2.58	23.65±4.50	23.83±4.51	16.51±2.47	15.95±2.59

**Table 4:** Comparison of dry bones and OPG available in the literature.

Author		MF-LB		MF-PB		MF-Notch		MF-AB	
		RT	LT	RT	LT	RT	LT	RT	LT
**Patil et *al***. [20]	Dry	-	-	-	-	13.33±1.57		23.67±3.45	24.36±2.31
	OPG	-	-	-	-	13.73±1.73		25.59±4.33	27.48±2.97
**Moudi et *al***. [24]	Dry	-	-	-	-	-	-	-	-
	OPG	23.81±4.49		12.78±2.88		27.64±6.03		-	-
**Present study**	Dry	23.85±4.21	24.81±4.77	13.51±2.10	14.16±2.27	23.44±3.86	23.05±3.99	16.41±2.42	16.18±2.47
	OPG	27.26±4.37	27.19±4.93	14.46±2.46	15.11±2.58	23.65±4.50	23.83±4.51	16.51±2.47	15.95±2.59

## DISCUSSION

### Visibility

In our study, out of 200 OPG, MF was bilaterally invisible on eight radiographs, unilaterally on right side in four OPG and on the left side in five OPG. In a study conducted by Soheilifar et al. [11], researchers found it was invisible bilaterally in three cases. The invisibility of mandibular foramen has been attributed to the density of bone by Afkhami et al. [12].

### MF-PB

In the present study, the mean distance (MF-PB) on the right side was 13.51 mm with (SD±2.10 mm) on dry bones and 14.46 mm (SD±2.46 mm )on OPG, while on the left side, the mean distance was 14.16 mm (SD±2.27 mm) on dry bones and 15.11 mm (SD±2.58 mm) on OPG. It is evident that the actual distance was less than it appeared on OPG (Table 4). Knowing about the percentage of measurement error can play a crucial role during procedures or before planning treatment in this region. MF is located farther away from the posterior border of the ramus on the left side than on the right side (Table 1). Other authors [5, 6, 9, 13–15] also found the distance greater on the left side than on the right side, except for some studies [7, 16–18]. A statistically significant difference (P=0.009) was found between the two sides of dry bones.

Similarly, comparing dry bone and OPG, a highly statistically significant difference was found on both sides (RT, p=0.0001 and LT, p=0.0002). Values in the current study vary from those observed by previous authors (Tables 3 and 4). The difference may be attributed to racial factors, ethnic variations, and dietary habits. Magnification on the right side was 7.03% and 6.71% on the left side. In an intraoral vertical split osteotomy, a cut is given at a distance of 7–8 mm from the posterior border of the ramus of the mandible. So, it is important to know the location of the mandibular foramen from the posterior border of the ramus to avoid neurovascular injury.

### MF-AB

In this study on dry bones, from the anterior border of the ramus of the mandible, MF was found to be at a mean distance of 16.41 mm (SD±2.42 mm) and 16.18 mm (SD±2.47 mm) on the right and left sides, respectively. It was 16.51 mm (SD±2.47 mm) and 15.95 mm (SD±2.59 mm) on OPG on the right and left sides, respectively. Hence, it is clear that the distance measured on OPG on the left side was less than on dry bone, so OPG is not reliable for this parameter (P=0.03). If we compare the right and left sides of dry bones, no statistically significant difference was found (P=0.55). The present study findings agreed with Nivedha et al. [9], Oguz and Bozkir [13], Kumari S. et al. [15], and Hoque et al. [17]. The percentage of magnification on the right side was 0.60%, and -1.42% on the left side. It can be stated from the results that the ramus is compressed on OPG from its anterior border. A modified IANB technique by Thangavehi et al. [14] suggested inserting a needle at a distance of 8–10 mm from the anterior border of the ramus. To assess the depth of needle insertion in the region of pterygotemporal depression, a dentist approaches the mandibular foramen from the anterior border of the ramus. It is considered that the inferior alveolar nerve moves 4 mm posteriorly from its location when a patient opens his/her mouth.

### MF-Notch

The mean distance of the mandibular foramen to the mandibular notch was 23.44 mm (SD±3.86 mm) and 23.05 mm (SD±3.99 mm) on the right and left sides, respectively. On OPG, it was 23.65 mm (SD±4.50 mm) and 23.83 mm (SD±4.51 mm) on the right and left sides. There was no statistically significant distance between dry bone and OPG. Our findings were close to Prado et al. [16] and Reddy et al. [19]. Magnification on the right was reported as 0.86% and on the left side as 3.38%.

### MF-LB

The mean distance between the mandibular foramen and the lower border of the mandible was 23.85 mm (SD±4.21 mm) on the right side and 24.81 mm (SD±4.77 mm) on the left side. On OPG, it was recorded as 27.26 mm (SD±4.37 mm) and 27.99 mm (SD±4.93 mm) on the right and left sides. There was a highly statistical difference between dry bones and OPG. Findings in the present study were lower compared to other studies available in the accessible literature. Magnification on the right side was 14.30% and 12.82% on the left side.

The study conducted by Patil et al. [20] agreed with the current study on finding a statistically significant difference between measurements on dry bones and OPG. Their study found a significant difference between the measured distance except between the anterosuperior point of MF to the mandibular notch. A statistically significant difference can be attributed to the angulation of the panoramic machine and the three-dimensional structure of the mandible. In the present study, a statistically positive correlation was found between dry bones and OPG, which was in line with others [20, 21]. A study conducted by Appana et al. [10] reported the error of magnification as 22.08%.

Morphometric localization of mandibular foramen concerning anatomical landmarks would help innovate new instruments and advance techniques [14]. If any unfavorable situation occurs during surgeries, awareness of various distances may help a surgeon modify procedures to avoid complications and disturbance of vital structures [22]. Distortion and magnification of OPG are not uniform, and it varies from one region to another of a single structure. Therefore, before proceeding with any treatment, knowledge about quantification and distortion depending upon the region is of utmost importance.

## CONCLUSION

The current findings show a statistically significant difference regarding distances between dry bone and OPG but no statistically significant difference between MF-notch on both sides and MF-AB on the right side. As a result, a surgeon can rely upon a mandibular notch to locate mandibular foramen during clinical procedures. OPG does not provide pinpoint accuracy; for precise localization, it is always advisable to proceed with advanced three-dimensional techniques to protect viable anatomical structures. Although OPG has magnification and distortion, the distance of the mandibular foramen from the anterior border of the ramus of the mandible was shorter on OPG than on dry bone in the present study. As a result, in developing countries where OPG is most widely utilised, this distance must be considered. Exploring cost-effective procedures with less radiation exposure can be enticing.
